# Drone approach parameters leading to lower stress sheep flocking and movement: sky shepherding

**DOI:** 10.1038/s41598-021-87453-y

**Published:** 2021-04-08

**Authors:** Kate J. Yaxley, Keith F. Joiner, Hussein Abbass

**Affiliations:** 1 School of Engineering and IT, University of New South Wales (UNSW), Canberra, Australia; 2 Capability Systems Centre, UNSW, Canberra, Australia; 3 School of Engineering and IT, UNSW, Canberra, Australia

**Keywords:** Zoology, Engineering, Mathematics and computing

## Abstract

Consumer groups are pressuring modern farmers to be more efficient with a focus on better animal welfare. Herding risks farmer lives, involves stress from farm dogs, and if not performed often and intelligently, risks neglect. We examined the behavioural and physiological response of twelve Dorper sheep (*Ovies aries*) to a drone to adapt mathematical models of shepherding to the new dimension. The model aims to make it feasible for artificial intelligence to improve the autonomy of farmers and pilots in shepherding from the sky. Sheep acclimatised quickly and positively to the drone initiating drive of a flock, regardless of drone speed. Our results demonstrate that stimulating sheep auditory awareness during herding from the sky leads to varying sheep responses. When controlled, these auditory cues can maintain safer distances between the drone and the sheep, offering great potential for the agriculture industry. We outline our ongoing research plans to achieve more autonomous sky shepherding that is compassionate to animal welfare and trusted by farmers and the consuming public.

## Need for improved shepherding in the agricultural industry

In Australia, like in many developed countries, public consumers are pressuring modern farmers to provide better animal welfare and be more efficient—so-called smart farming^1^. There are now larger farms managed by fewer farm workers using more machinery^2^. For sheep and cattle production, large farms result in bigger areas and more animals to survey, increasing the distances farmers and working dogs must travel using motorbikes or quad bikes^3^. Farmer deaths from accidents are significant^4,5^, while research on sheep welfare has called for new practical technologies to assist in the detection of multiple welfare issues^6^.

Use of working dogs to herd animals’ risks consumer concerns about the stress on the animals, incidents of biting and pressure to muzzle dogs^7^. Heart rate is a generally accepted indicator of stress in sheep that is ideally kept in a range around 163 beats per minute average when being vigorously driven or 262 beats per minute instantaneous for acceptable stress in the presence of working dogs; where, by comparison, the resting heart rate for sheep is around 80 beats per minute^8^.

Drones offer farmers new dimensions in access and robotics^1,9^. Sheep farmers in countries like New Zealand have resorted to using piloted drones to herd^10^ however, research of the effect on animal stress and other behavioural responses has not been conducted. Drones have been successfully used to herd wild elephants and birds^11,12^, with Hahn et al.^11^ observing for elephants that ‘*As the drone approached, herds would group together quickly and flee rapidly as the drone came within c. 50 m of the closest individual*’ with pilots ‘*able to control the movement of the elephants through herding tactics, positioning the drone on either flank of the herd.*’ There is the clear potential to use drone technology for shepherding and a need to underpin that use with scientific research.

## Preparing for herding with autonomous artificial intelligence (AI)

Public videos^10^ show there is considerable piloting skill required in using drones close to animals and the ground, that when coupled with the skills and awareness of shepherding and complex farm terrain, could be costly. Mustering cattle with helicopters in the Australian outback, however, has proven cost-effective despite the complexity^13^. In order to improve on that piloted approach, our ultimate research goal is autonomous shepherding with AI and to do so while engendering public and farmer trust.

Early work on herding behaviour proposed that herding is motivated by self-preservation rather than groupthink and overall group protection^14^. Mathematical models for herding developed based on three vector relationships of repulsion, alignment, and attraction^15^. These models were extended to develop a ground-based simulation for the reactions of a sheep in response to a sheepdog using the repel and attract vectors directly and align vector indirectly^16^. The Strömbom Model of herding^16^ is based on rule-based switching between collecting sheep and driving them to a goal. Sky-based models of shepherding have not been validated. Moreover, shepherding models have so far ignored the effect of noise such as dogs barking, motorbikes approaching, or for this research, a drone approaching and emitting noises from a speaker. Sheep have excellent hearing^17^ and eyesight^18^. A trial of merino sheep^19^ found ‘*that sheep can perform a discriminant, operant task based on a visual cue … and indicates the potential for sheep to use audio cues in their learning.*’

**Figure 1:**
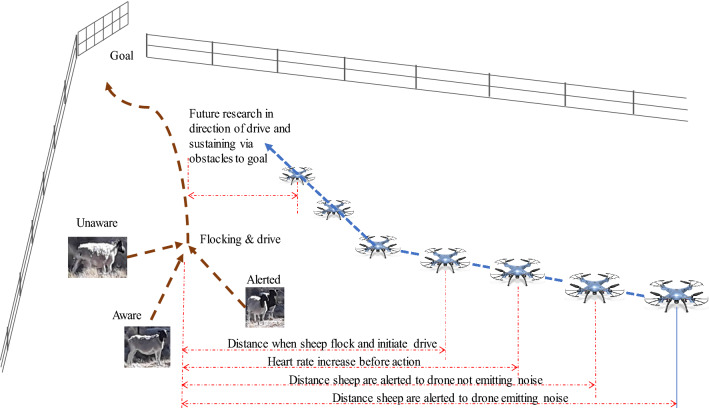
Expected proximal response of sheep to the presence of a Sky Shepherd.

Our context is the response of sheep to a drone (Fig. 1) and we hypothesise that involves sheep being alerted by the noise of the drone, followed by sensing the drone’s physical presence, then to physiological responses to the perceived threat with increased heart rate^20,21^, before finally to cluster and drive away from the drone.

**Figure 2:**
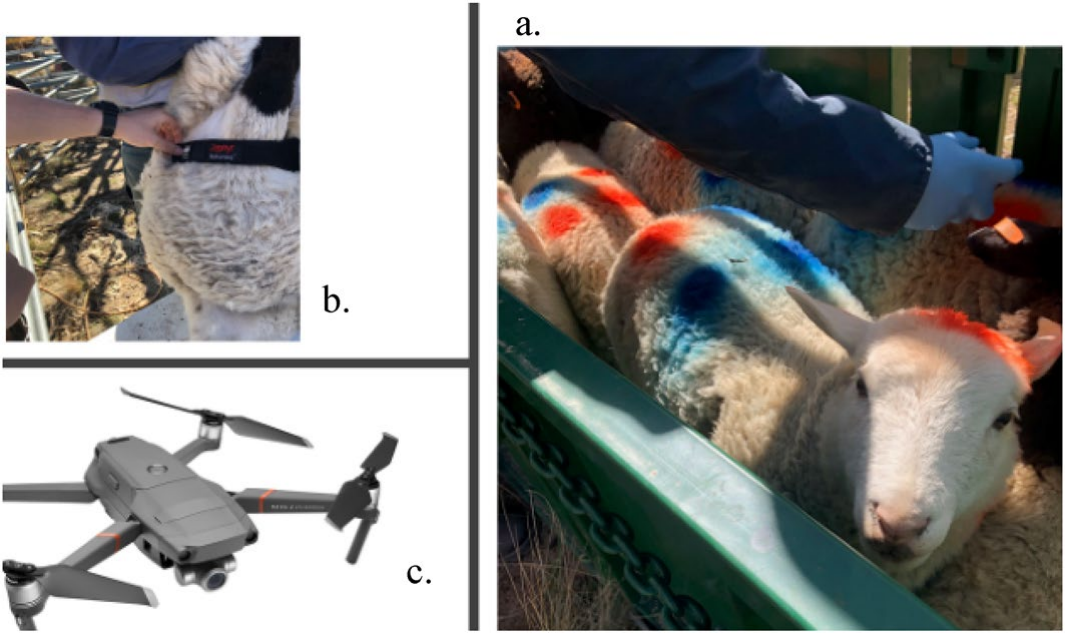
Monitoring Dorper sheep health and welfare.

The sheep’s health and welfare were monitored largely through visual and heart-rate observations. Individual sheep were monitored using dye markings (Fig. 2a.), while their heart rate was monitored using a Zephyr bio-harness (Fig. 2b.). We explored what were the least stress, and tighter flock formations for a shepherding drone, by reviewing video footage and behaviours of the sheep throughout the experiment process (Fig. 2c.).

Shepherding is challenging due to its extremely large degrees of freedom in the state and action spaces of sheep and sheepdog. Attempts in artificial intelligence to learn the state-action maps combined mathematical models with a need to sub-divide and order the behavioural space of the sheepdog to make machine learning more assured using a curriculum-based approach^22^. Interactive simulation is used to generate demonstrations for the machine to learn from. For this ‘*imitation learning*’^23^ approach, we needed to first examine sheep behavioural and physiological responses to the position and noise of a drone.

## Methods

**Figure 3:**
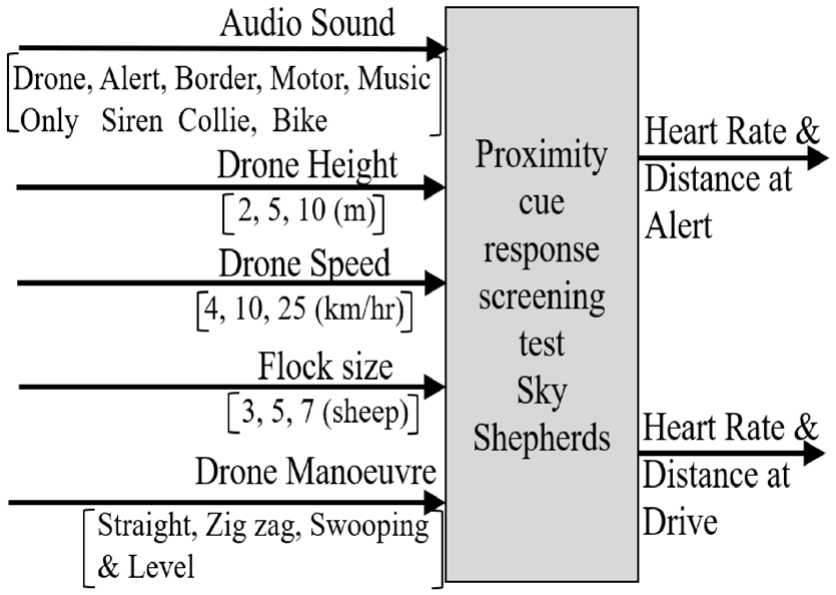
Research inputs and outputs.

The stimuli evaluated, the levels set, and the dependent outputs measured (Fig. 3) were evaluated using a high throughput test design. There were 18 test runs each repeated three times for variation over a two-week data collection period. We routinely mixed the sheep with randomization that was separate from any run order (allocation concealment), with small flock sizes of between three and seven sheep, which is at the lower limit of successful flocking behaviour found by Penning et al. with respect to grazing intakes^24^. Our testing was further limited by batch effects and a degree of sheep learning^19,25^.

### Ethical concerns

The University of New South Wales Animal Ethics Committee approved this research (ACEC 19/122B), and we complied with all conditions of our submission throughout the testing. Further, all methods are reported in accordance with ARRIVE guidelines (https://arriveguidelines.org).

### Animals

The sheep used were twelve Dorper sheep, characterised in three research studies in 2000 by Schoeman^26^, Brand^27^, and Milne^28^. The twelve animals were mixed wethers and ewes and unfortunately the farm used did not have scales to weigh each sheep. Our heart rate monitors were non-invasive strap-on ones (Fig. 2) with a small GPS locator added onto the band at the top. Heart rates measured were peak and instantaneous and not averaged unless for cross-run statistical comparison. We painted sheep on the back with a binary code using normal veterinarian marking dye so they could be individually identified (Fig. 2). Sheep were herded normally to a 250 m by 250 m paddock in small flocks between 0900 and 1630 h and allowed to settle with hay before being approached by the drone performing the test run. We stopped the test if the heart rate of sheep exceeded 200 peak beats per minute. The drone was not allowed to enter the 20 m safety box around the sheep. A spot of dye was also put on each sheep’s head to help identify if it was lowered and eating or raised and alert. By the second test of day one of testing (03 Sep 2019), each sheep had been exposed to the drone. At completion of the 54 runs, the minimum exposure event was 21.

### Test design

The decision to screen the main effects before doing deeper analysis follows the experience of the Six Sigma movement in exploring combinatorial possibilities^29^. The screening conducted is shown in Fig. 3, where the independent or input variables to be evaluated are on the left and the dependent variables or outputs, possibly affected by the input variables, are on the right. The design of the tests was a highly efficient combinatorial method known as high throughput testing, used in pharmaceutical^30,31^, genetics^32^, material science^33^ and software functionality^34,35^. A test design package was used that compares all possible test design combinations and presents the minimum number of tests while maximising the independence of each input on the output (i.e., orthogonality). The result was 18 test runs, shown in Table 1, each repeated three times to assess variation and if learning was occurring: a total of 54 separate tests. By comparison, a full factorial design would have required 405 test runs, that when repeated for variation would have been 1215 individual test events.

**Table 1:** Test design and conduct.

	Rep	Flock composition	Date of test
**Serial 1** Sound = off; Profile = Straight and level; Height = 10 m; Speed = 4 km/h	1	23*, 24*, 30, 41, 43*, 51	03 Sep 2019
	2	24, 30*, 41, 43, 52*, 54*	10 Sep 2019
	3	23*, 30, 42*, 43*, 47, 52	12 Sep 2019
**Serial 2** Sound = off; Profile = Straight and level; Height = 2 m; Speed = 10 km/h	1	34*, 42*, 52*	03 Sep 2019
	2	43*, 47*, 50*	11 Sep 2019
	3	24*, 34*, 54*	12 Sep 2019
**Serial 3** Sound = off; Profile = Zig zag; Height = 5 m; Speed = 10 km/h	1	30*, 47, 50*, 51*, 54	03 Sep 2019
	2	23, 34*, 47*, 50, 51*	10 Sep 2019
	3	23*, 30*, 42*, 47, 52	12 Sep 2019
**Serial 4** Sound = off; Profile = Zig zag; Height = 2 m; Speed = 25 km/h	1	23*, 42*, 52*	03 Sep 2019
	2	24*, 51*, 54*	11 Sep 2019
	3	23*, 47*, 50*	11 Sep 2019
**Serial 5** Sound = off; Profile = Swooping; Height = 10 m; Speed = 25 km/h	1	23, 30*, 34, 41*, 43, 52*	06 Sep 2019
	2	23*, 30, 41*, 42*, 47, 52	12 Sep 2019
	3	24, 30*, 41, 43, 52*, 54*	10 Sep 2019
**Serial 6** Sound = off; Profile = Swooping; Height = 5 m; Speed = 4 km/h	1	42, 47*, 50*, 51, 54*	06 Sep 2019
	2	24, 41*, 42*, 51*, 52	11 Sep 2019
	3	23, 34*, 47*, 50, 51*	10 Sep 2019
**Serial 7** Sound = Siren; Profile = Straight and level; Height = 5 m; Speed = 10 km/h	1	23*, 24*, 34, 42, 43, 51*	06 Sep 2019
	2	30*, 34, 41*, 43*, 50, 54	10 Sep 2019
	3	23*, 30, 41*, 42*, 47, 52	12 Sep 2019
**Serial 8** Sound = Siren; Profile = Zig zag; Height = 2 m; Speed = 4 km/h	1	30*, 50*, 54*	06 Sep 2019
	2	24*, 34*, 54*	12 Sep 2019
	3	43*, 47*, 50*	11 Sep 2019
**Serial 9** Sound = Border collie; Profile = Straight and level; Height = 10 m; Speed = 25 km/h	1	24, 41, 42*, 43*, 51, 52*	06 Sep 2019
	2	23, 30*, 42*, 43, 47*, 50	11 Sep 2019
	3	23*, 24*, 34, 43*, 51, 54	12 Sep 2019
**Serial 10** Sound = Border collie; Profile = Zig zag; Height = 10 m; Speed = 4 km/h	1	30, 34*, 47*, 50, 54*	06 Sep 2019
	2	24, 34, 51*, 52*, 54*	11 Sep 2019
	3	41*, 42, 47, 50*, 52*	12 Sep 2019
**Serial 11** Sound = Border collie; Profile = Swooping; Height = 2 m; Speed = 4 km/h	1	24*, 41*, 51*	06 Sep 2019
	2	43*, 47*, 50*	11 Sep 2019
	3	23*, 47*, 50*	11 Sep 2019
**Serial 12** Sound = Border collie; Profile = Swooping; Height = 5 m; Speed = 25 km/h	1	24*, 34*, 43*	09 Sep 2019
	2	34*, 41*, 50*	12 Sep 2019
	3	43*, 47*, 50*	11 Sep 2019
**Serial 13** Sound = Motorbike; Profile = Straight and level; Height = 5 m; Speed = 4 km/h	1	24, 30*, 34*, 42, 43, 50*	09 Sep 2019
	2	23*, 30, 42*, 43*, 47, 52	12 Sep 2019
	3	23, 30*, 42*, 43, 47*, 50	11 Sep 2019
**Serial 14** Sound = Motorbike; Profile = Zig zag; Height = 5 m; Speed = 25 km/h	1	23, 47*, 51*, 52*, 54	09 Sep 2019
	2	41*, 42, 47, 50*, 52*	12 Sep 2019
	3	24, 34, 51*, 52*, 54*	10 Sep 2019
**Serial 15** Sound = Motorbike; Profile = Swooping; Height = 2 m; Speed = 10 km/h	1	24, 30, 34*, 41*, 42, 43*	09 Sep 2019
	2	23*, 24*, 34, 43*, 51, 54	12 Sep 2019
	3	30*, 34, 41*, 43*, 50, 54	10 Sep 2019
**Serial 16** Sound = Motorbike; Profile = Swooping; Height = 10 m; Speed = 10 km/h	1	24, 30*, 41, 42*, 52*	09 Sep 2019
	2	24*, 34*, 41, 51, 54*	11 Sep 2019
	3	24, 41*, 42*, 51*, 52	11 Sep 2019
**Serial 17** Sound = Music; Profile = Straight and level; Height = 2 m; Speed = 25 km/h	1	23, 47*, 50, 51*, 54*	09 Sep 2019
	2	23*, 30*, 42*, 47, 52	12 Sep 2019
	3	24*, 34*, 41, 51, 54*	11 Sep 2019
**Serial 18** Sound = Music; Profile = Zig zag; Height = 2 m; Speed = 25 km/h	1	23*, 51*, 54*	09 Sep 2019
	2	24*, 34*, 51*	12 Sep 2019
	3	24*, 51*, 54*	11 Sep 2019

*Indicates Zephyr Bioharness instrumentation.

The test design package is one by Phadke Associates called rdExpert Test Planning Lite version 10.6.02. The algorithmic optimiser the package uses to generate the minimum number of all two-way combinations of the factors and levels/categories is proprietary; however, we checked the test design output for its rigour and orthogonality before use. The test design table used is available in extended data.

### Experimental procedure

We used a total of 12 Dorper sheep that we randomly mixed for different flocks and heart rate monitoring. For each of the test runs, we generated flocks as per the test design and three sheep in that flock we fitted with Zephyr Bioharness 3.0 heart rate monitors with a separate GPS unit of QStarz BT-Q818XT. The accuracy of the GPS was around 3 m. Correct reading was established before sheep were mustered to the test paddock, where we positioned some Lucerne hay to help them settle^21^. We monitored the heart rates until these settled to the resting rates observed by^8^; usually about 10–15 min. The drone was a DJI Mavic 2 Enterprise Duo with an included speaker. Our drone GPS we provided separately using a Zephyr bio-module and QStarz BT-Q818XT. We flew the drone to an initial designated point 200 m from the sheep and then made the designated approach for the test run number. As soon as sheep had flocked together and began to drive, or if the minimum safety box of 20 m from the sheep was achieved, we ceased the drone approach. Each time after the first full set of 18 runs, that is during repetition one, the sheep flock was shuffled among the available sheep, and new sheep were instrumented for the next run. For repetitions two and three, we rested each flock in the test paddock after a test run from repetition two and gave them a different test run from repetition three. The drone direction was also varied slightly.

### Observational analysis

The video from the drone was fully time-captured and analysed afterwards to determine the drone’s distance from the sheep at which alert occurred, denoted by the lifting of the head from feeding, looking towards the drone and if possible, turning of the ears. We continued to analyse the video to identify the distance of the drone from the sheep when the sheep flocked and began to drive away from the drone. We excluded 16 of the 54 tests at the alert condition mainly due to occasional drop out of drone video and positional information at longer ranges. These dropouts did not affect the drive condition. An extended data file is available for this research.

### Statistical analysis

We used Quantum Excel (XL) (2016) [Version 5.29.1700] to perform our statistical analyses. Quantum XL is an add-on package with a Design Of Experiments (DOE) capability for multi-factor, multi-input analysis in the analysis style of Six Sigma^36^. The test runs were first run as historical data analysis, meaning that the five input factor, four outputs, 18 test runs with three repetitions by rdExpert is non-standard. Multi-factor linear regressions were conducted with only main effects and no interactions loaded, principally because the orthogonality of the combinatorial test design is only ‘*nearly-orthogonal*’ meaning it has a variance inflation factor ≤ 1.4 and interactions are not adequately orthogonal or resolvable from such sparse screening designs^29^. The limited orthogonality was exacerbated by the categorical factors, such as *broadcast noise*, where each of the five categories has to be assessed against the other categories. We generated main effects plots to indicate the direction of high-low comparisons for each factor or between categories. Where we found significance, we then conducted an individual statistical comparison between levels or categories. The multi-factor linear regressions were examined both for the mean of each of the four outputs and the standard deviation, a total of eight regressions. The probabilistic modelling completeness of these regressions were non-existent to weak as there were many unmodelled factors; it was primarily a means to establish potential effects for further analysis.

To examine the effect of batching^32,36^, we examined for statistically significant differences across repetitions for each of the four outputs. As there were significant differences between the repetitions for the drive condition, attributed to sheep acclimatising, much of the focus shifted to comparing the effects of the evolving factors from repetition one to three.

Each time we performed a statistical significance test, we first checked comparative outputs for normality. If the result for all outputs were normal, then we performed an F-test to check for equal variance, then the t-test performed with equal or unequal variance as appropriate. If any of the outputs were not normal, we performed the non-parametric Mann–Whitney test for median differences and checked variation using Levene’s test. On some occasions, the Mann Whitney test revealed the existence of likely outliers, and in these instances, we used Mood’s Median test to be conservative. Significance was generally taken at the 95 per cent confidence, though as this was screening involving animals with significant variation, 90 per cent likelihood of significance was occasionally noted. Statistical notes are provided at the end of this article.

## How sheep responded and low-stress options

Our results are given first for sheep being alerted to the drone, the alert condition, and then when sheep collected and began driving, the drive condition. We ceased only three of our test runs for safety reasons. The peak heart rates throughout drone testing were consistently less than the peak emplacement heart rates using a dog or motorbike to bring the sheep to the testing arena.

### Alert

We found no evidence of the sheep’s alert responses changing significantly across the three repetitions. Sheep were alerted to the drone much earlier if the additional sound was broadcast. Across all testing, the distance at which sheep were alerted improved significantly from a mean of 51 m with just drone engine noise to a median of 82 m when broadcasting any of the four sound types trialled (p < 0.002), with no significant increase in heart rate. As such, broadcasting sound maintains a larger distance between the drone and the sheep; therefore, it is safer and more effective.

**Figure 4:**
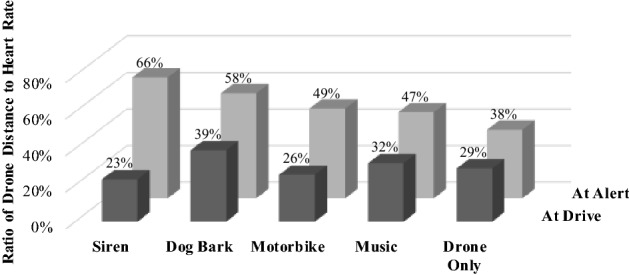
Tradeoff for different sound effects between good distance and low heart rate.

To choose which sound is best to alert sheep is a tradeoff between stress, as measured by heart rate, and effectiveness, as measured by distance from the drone. The tradeoff is illustrated in Fig. 4 by the ratio of the distance between the drone and sheep and the heart rate at the time of sheep being alerted (back row) and at time of sheep beginning to drive (front row). The sound of an alert siren is the best tradeoff at alert, while the sound of dog bark is the best tradeoff at drive. A more detailed probabilistic modelling and optimisation confirmed the best trade-off.

We found drone manoeuvre, speed or height and flock size were not significant factors in alerting sheep, at least across repetitions and within the ranges tested. As there is no advantage in drone manoeuvre, the safer and more economical straight and level flight is preferred. A minor exception to note in the first repetition was the heart rate of sheep was significantly more consistent when flying at 10 m height with a favourable negative skew around a mean of 141 beats per minute, compared to flying at 2 m or 5 m with unfavourable positive skew around a mean of 148 beats per minute (p = 0.016). We attribute this better consistency in our stress measurement to the certainty of better visibility at this height compared to lower heights; thus, there is a benefit in not surprising sheep on approach.

In summary, a shepherding drone should ideally approach to alert its presence with the *siren* broadcasting at the height of 10 m and flying straight-and-level. In doing so, our hypothesis is supported with higher predictability in sky shepherding tasks achieved.

### Initiating drive

We found sheep acclimatised quickly to the drone at the drive, with their heart rate dropping significantly from a mean of 162 beats per minute in the first repetition to 144 and 142 beats per minute in the later repetitions (p < 0.011). Reinforcing that acclimatising is occurring, the heart rate is more consistent after the first repetition (p < 0.07). We also found the distance from the drone that sheep began to drive improved from a median of 34 m in the first repetition to better than 53 m in later repetitions (p < 0.06), albeit with significantly increased variance (p = 0.007). We suggest there are generally positive but differential learning rates among the sheep. Given the heart rate finding, this response is without any increase in stress.

Like the alert findings, sheep begin driving significantly further from the drone when a sound is broadcast compared to the drone noise alone (p = 0.043) including likely more variance with a sound broadcast than without (p = 0.088). Our choice of the best sound to broadcast to initiate drive is again a trade-off (Fig. 4), where a dog bark initiates drive at an average distance of 64 m and an average heart rate of 164 beats per minute compared to less effective noise types like the alert siren at an average distance of 36 m and average heart rate of 158 beats per minute (p = 0.052). Factors other than sound were not significant, except for the following findings for the final repetition:

- Sheep had significantly lower heart rate when initiating drive at the higher drone speed of 25 km/h compared to the lower speeds (p < 0.087). High drone speed may initiate drive before stress has fully manifest and later evolutions in shepherding will need to examine this.
- Flying at 10 m height rather than lower heights likely reduces sheep heart rate and thus stress, likely due to the visual acuity of sheep in the horizontal and vertical planes^37^ (p = 0.096).
- Larger flocks are possibly less stressed than smaller flocks (p = 0.14), consistent with prior research^38^.

In summary, a drone should initiate drive with: (1) dog bark emitting, (2) any drone manoeuvre, (3) speed of 25 km/h, and (4) height of 10 m.

## Discussion

In comparing artificial neural networks to animal brains, Zador^25^ argues that ‘a large component of an animal’s behavioural repertoire is not the result of clever learning algorithms—supervised or unsupervised—but rather of behaviour programs already present at birth.’ Our hypothesis for the adaptation of the sheep to our new stimuli in sky shepherding is that the innate survival behaviours of flocking and flight, otherwise called predation^20^, have been triggered by the association to motorbike and dog barking sounds^21^. Another view may be that our inclusion of auditory cues to improve predictability and control^39^ during shepherding tasks has likely supported lower heart rate response and improved learning within the flock. This observation may be ‘transfer learning’ as proposed by Zador^25^ which he notes operates ‘not only within a single sensory modality like vision, but across sensory modalities.’ Overall, we have evolved the human-sheep construct^38^ to one that considers the welfare of the sheep by introducing an ability to improve predictability and control during necessary shepherding tasks.

**Figure 5:**
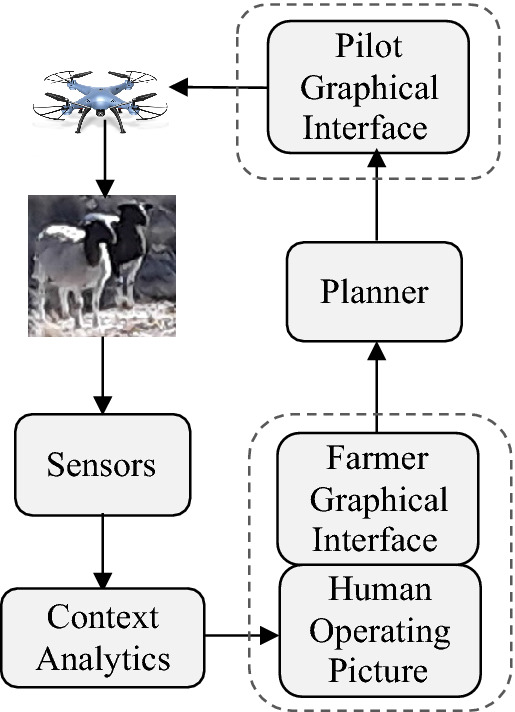
Sky shepherd human-autonomy team AI.

To enable our approach to progress and implement improved human-autonomy-animal teaming, our answer is to create a collaboration and combination of robots, farmers and pilots (Fig. 5)^23^, where humans, the human experience of working dogs, and sheep innate behaviours will form simplifying rule sets and imitation learning to enable the AI and encode a welfare fostering approach. By using an iterative approach to introducing sky shepherding, our program will continue to foster a design approach that considers the welfare of the animal and human users in the system. Using similar approaches to training AI for autonomous sky shepherding, we will be able to fully realise what AI can offer to support improved practices.

The cognitive edge of shepherding has so far only been explored in human-led simulations for machine learning in two dimensions^22^, but the multi-dimensional sheep-response model will enable this work to be more representative and lead to more AI-led fieldwork. The aim remains as per Fig. 1, with a desire to reduce task completion time, sheep stress level, and increase the mastery-level of humans teaming with the AI. In between the farmer and the human pilot resides the AI-based decision support system that transforms the farmer’s intents into low-stress and ideal waypoints for the pilot to follow. This human-autonomy teaming should address the necessary AI ethical principles of transparency, non-maleficence, and beneficence^40^ through the necessary design considerations.

## Conclusion and future work

Our research has shown that combining aural cues with drones could improve safety without stressing the animal. The research offers farmers the prospect of being both more efficient and caring better for their sheep to achieve smart farming^1^. Our next research challenge will involve developing mathematical models to capture the modulation of the drone’s influence vectors during longer driving and collecting tasks. The testing will also explore dealing with obstacles such as fences and trees and whether early predictive work on the repulsion force and distances between sheep and obstacles is representative (Supplementary Information S1).

### Statistical finding notes

Key statistical findings and the analysis used are as follows, in the order presented in the paper:

- Across all testing the distance at which sheep were alerted improved significantly when broadcasting any of the four sounds [Mann–Whitney *U* = 124, df = 1, n_1_ = 11, n_2_ = 27, p < 0.002] with no significant increase in heart rate at alert from broadcasting sounds [Mood’s ${\chi }^{2}$ = 2.37, df = 1, n_1_ = 18, n_2_ = 36, p = 0.12).
- The sound of an *alert siren* is the best tradeoff at alert. A multiple linear regression was calculated to predict heart rate (beats/min) and distance (m) at the alert condition, based on the independent variables in Fig. 3. Non-significant regression equations were found for both the mean of heart rate (F(9, 44) = 0.4388, p = 0.9065, R^2^ = 0.0824) and distance (F(9, 28) = 1.7088, p = 0.1338, R^2^ = 0.3545 and their standard deviations (F(9, 8) = 0.8832, p = 0.5751, R^2^ = 0.4984; F(9, 3) = 2.1455, p = 0.2869, R^2^ = 0.8655 respectively). When optimising the regression model in *QuantumXL* to maximise the distance and constraining the heart rate to less than 130 beats/min, the independent variable levels for optimum are a sound of *alert siren*, *straight and level* flight, *drone height* of 10 m, *drone speed* of 4 km/h and *flock size* of seven sheep. The indicative non-significant prediction at that optimum is M = 127.8 beats/min, SD = 11.9 beats/min, M = 89.8 m, SD = 37.2 m.
- The sound of *dog bark* is the best tradeoff at drive. A multiple linear regression was calculated to predict heart rate (beats/min) and distance (m) at the drive condition, based on the independent variables in Fig. 3. Non-significant regression equations were found for both the mean of heart rate (F(9, 44) = 0.9276, p = 0.5110, R^2^ = 0.1595) and distance (F(9, 44) = 0.9053, p = 0.5291, R^2^ = 0.1562 and their standard deviations (F(9, 8) = 02.8537, p = 0.0773, R^2^ = 0.7625; F(9, 8) = 1.8175, p = 0.2061, R^2^ = 0.6716 respectively). When optimising the regression model in *QuantumXL* to maximise the distance and constraining the heart rate to less than 150 beats/min, the independent variable levels for optimum are a sound of *dog bark*, *zig zag* flight, *drone height* of 2 m, *drone speed* of 4 km/h and *flock size* of seven sheep. The indicative non-significant prediction at that optimum is M = 144.0 beats/min, SD = 15.2 beats/min, M = 71.6 m, SD = 61.8 m.
- The heart rate of sheep was significantly more consistent when flying at 10 m height compared to flying at 2 m or 5 m [10 m *height* (M = 141 beats/min, SD = 8 beats/min) c.f. 2 m and 5 m *heights* (M = 148 beats/min, SD = 23 beats/min; conditions F(16) = 0.124, p = 0.016, one-sided, n_1_ = 6, n_2_ = 12].
- Sheep acclimatised to the drone at the drive, with their heart rate dropping significantly across repetitions and with more consistency [Repetition 1 M = 162, SD = 24 c.f. Repetition 2 and 3, M = 144 and 142, SD = 15; conditions t(34) = 2.7 and 3.1, p = 0.011 and 0.004; F(34) = 2.6 and 2.5, p = 0.06 and 0.07]
- Sheep acclimatised to the drone at the drive, with the distance they began drive increasing significantly across repetitions albeit with significantly less consistency [Repetition 1 Mdn = 34 m, SD = 17.7 m; Repetition 2, Mdn = 44 m, SD = 22.8 m; Repetition 3, Mdn = 53 m, SD = 34.6 m; Mann–Whitney R1 to R2 *U* = 270, p = 0.048; R1 to R3, *U* = 273, p = 0.0598; R2 to R3, *U* = 319, p = 0.6693; and Levene’s L = 5.47, p = 0.007, DF = 2]
- Sheep begin driving significantly further from the drone when a sound is broadcast compared to when it is not, including the likelihood of more variance with a sound broadcast than without [No sound Mdn = 33 m, SD = 22.9 m, with sound Mdn = 45 m, SD = 28.7 m; Mann Whitney U = 401, df = 1, n_1_ = 18, n_2_ = 36, p = 0.043; Levene’s L = 3.03, p = 0.088]
- Broadcasting a *dog bark* initiates drive at significantly higher distance than does broadcasting an *alert siren*. [*Dog bark*, M = 64 m c.f. *Alert Siren*, M = 36 m; t(17) = 2.12, df = 1, n_1_ = 12, n_2_ = 6, p = 0.052]
- Sheep had significantly lower heart rate when initiating drive at the higher *drone speed* of 25 km/h compared to the lower speeds [Speeds at 25 km/h c.f. 4 km/h and 10 km/h; Mdn = 133 c.f. 154 and 144, Mann–Whitney *U* = 48 and 49, n_1_ = n_2_ = n_3_ = 6, p = 0.087 and 0.064 one-sided]
- Flying at 10 m *height* rather than lower heights likely reduces sheep heart rate and thus stress [*Height* 10 m, M = 135 b/min, SD = 19 b/min c.f. 2 m and 5 m, M = 145 b/min, SD = 13 b/min; conditions t(16) = (− 1.4), p = 0.096, one sided, n_1_ = 6, n_2_ = 12]
- Larger flocks of seven are possibly less stressed than smaller flocks [*Flock* of 7, M = 136 b/min, SD = 19 b/min c.f. flocks of 3 and 5, M = 145 b/min, SD = 13 b/min; conditions t(16) = (− 1.1), p = 0.14, one sided, n_1_ = 6, n_2_ = 12].

## Supplementary Information

Supplementary Information
