# Supplementary material for: Drone approach parameters leading to lower stress sheep flocking and movement: sky shepherding
Source: Sci Rep. 2021 Apr 8;11:7803. doi: 10.1038/s41598-021-87453-y (PMC8032684; doi:10.1038/s41598-021-87453-y)
Supplement: Supplementary file 1 — Supplementary Information [file 41598_2021_87453_MOESM1_ESM.docx]

**EXTENDED DATA**

| Run No. | Sound | Drone Manouevre | Drone Height (m) | Drone Speed (km/hr) | Flock size No. sheep |
| --- | --- | --- | --- | --- | --- |
| 1 | Drone only | Straight and level | 2 | 10 | 7 |
| 2 | Drone only | Straight and level | 10 | 4 | 3 |
| 3 | Drone only | Zig zag | 5 | 10 | 5 |
| 4 | Drone only | Zig zag | 2 | 25 | 3 |
| 5 | Drone only | Swooping | 5 | 4 | 7 |
| 6 | Drone only | Swooping | 10 | 25 | 5 |
| 7 | Alert Siren | Zig zag | 2 | 4 | 7 |
| 8 | Alert Siren | Straight and level | 5 | 10 | 3 |
| 9 | Dog bark | Straight and level | 10 | 25 | 7 |
| 10 | Dog bark | Zig zag | 10 | 4 | 5 |
| 11 | Dog bark | Swooping | 2 | 4 | 3 |
| 12 | Dog bark | Swooping | 5 | 25 | 3 |
| 13 | Motorbike | Zig zag | 5 | 25 | 7 |
| 14 | Motorbike | Straight and level | 5 | 4 | 5 |
| 15 | Motorbike | Dynamic | 10 | 10 | 7 |
| 16 | Motorbike | Dynamic | 2 | 10 | 5 |
| 17 | Music | Straight and level | 2 | 25 | 5 |
| 18 | Music | Zig zag | 10 | 10 | 3 |

*Table 1: Screening Test design of input factors (independent variables)*

| Run No. | Repetition 1 (beats/min) | Repetition 2 (beats/min) | Repetition 3 (beats/min) | Average (beats/min) | Standard Deviation (beats/min) |
| --- | --- | --- | --- | --- | --- |
| 1 | 124.00 | 142.00 | 142.00 | 136.00 | 10.39 |
| 2 | 127.00 | 141.00 | 136.00 | 134.67 | 7.09 |
| 3 | 133.00 | 145.00 | 145.00 | 141.00 | 6.93 |
| 4 | 154.00 | 135.00 | 140.00 | 143.00 | 9.85 |
| 5 | 143.00 | 137.00 | 144.00 | 141.33 | 3.79 |
| 6 | 140.00 | 161.00 | 128.00 | 143.00 | 16.70 |
| 7 | 133.00 | 158.00 | 138.00 | 143.00 | 13.23 |
| 8 | 122.00 | 128.00 | 147.00 | 132.33 | 13.05 |
| 9 | 147.00 | 157.00 | 134.00 | 146.00 | 11.53 |
| 10 | 146.00 | 113.00 | 139.00 | 132.67 | 17.39 |
| 11 | 147.00 | 124.00 | 145.00 | 138.67 | 12.74 |
| 12 | 176.00 | 139.00 | 131.00 | 148.67 | 24.01 |
| 13 | 152.00 | 145.00 | 155.00 | 150.67 | 5.13 |
| 14 | 143.00 | 148.00 | 153.00 | 148.00 | 5.00 |
| 15 | 149.00 | 115.00 | 91.00 | 118.33 | 29.14 |
| 16 | 205.00 | 127.00 | 143.00 | 158.33 | 41.20 |
| 17 | 143.00 | 145.00 | 122.00 | 136.67 | 12.74 |
| 18 | 138.00 | 134.00 | 157.00 | 143.00 | 12.29 |

*Table 2: Heart Rate data at Distance of Alert*

| Run No. | Repetition 1 (beats/min) | Repetition 2 (beats/min) | Repetition 3 (beats/min) | Average (beats/min) | Standard Deviation (beats/min) |
| --- | --- | --- | --- | --- | --- |
| 1 | 131.00 | 142.00 | 149.00 | 140.67 | 9.07 |
| 2 | 142.00 | 141.00 | 139.00 | 140.67 | 1.53 |
| 3 | 134.00 | 145.00 | 158.00 | 145.67 | 12.01 |
| 4 | 143.00 | 135.00 | 140.00 | 139.33 | 4.04 |
| 5 | 139.00 | 128.00 | 144.00 | 137.00 | 8.19 |
| 6 | 149.00 | 169.00 | 129.00 | 149.00 | 20.00 |
| 7 | 159.00 | 158.00 | 132.00 | 149.67 | 15.31 |
| 8 | 156.00 | 126.00 | 151.00 | 144.33 | 16.07 |
| 9 | 189.00 | 154.00 | 135.00 | 159.33 | 27.39 |
| 10 | 143.00 | 113.00 | 148.00 | 134.67 | 18.93 |
| 11 | 149.00 | 127.00 | 149.00 | 141.67 | 12.70 |
| 12 | 176.00 | 153.00 | 131.00 | 153.33 | 22.50 |
| 13 | 178.00 | 145.00 | 155.00 | 159.33 | 16.92 |
| 14 | 211.00 | 147.00 | 143.00 | 167.00 | 38.16 |
| 15 | 158.00 | 161.00 | 101.00 | 140.00 | 33.81 |
| 16 | 205.00 | 145.00 | 167.00 | 172.33 | 30.35 |
| 17 | 189.00 | 169.00 | 122.00 | 160.00 | 34.39 |
| 18 | 173.00 | 138.00 | 157.00 | 156.00 | 17.52 |

*Table 3: Heart Rate data at Distance of Initiating Drive*

| Run No. | Repetition 1 (m) | Repetition 2 (m) | Repetition 3 (m) | Average (m) | Standard Deviation (m) |
| --- | --- | --- | --- | --- | --- |
| 1 | 31.68 | - | - | 31.68 | N/A |
| 2 | 44.44 | - | 33.51 | 38.98 | 7.73 |
| 3 | 107.15 | - | 110.93 | 109.04 | 2.67 |
| 4 | 21.51 | - | - | 21.51 | N/A |
| 5 | 63.14 | 37.73 | - | 50.43 | 17.97 |
| 6 | 37.75 | 44.82 | 31.81 | 38.13 | 6.51 |
| 7 | 69.82 | - | 103.55 | 86.69 | 23.85 |
| 8 | 103.65 | 98.98 | 45.99 | 82.87 | 32.03 |
| 9 | 81.82 | 89.86 | 107.23 | 92.97 | 12.99 |
| 10 | 68.88 | - | - | 68.88 | N/A |
| 11 | 59.27 | 79.28 | 135.82 | 91.46 | 39.71 |
| 12 | - | 81.61 | 94.10 | 87.86 | 8.83 |
| 13 | 68.03 | - | - | 68.03 | N/A |
| 14 | 107.57 | 89.38 | 76.30 | 91.09 | 15.70 |
| 15 | 66.94 | 102.45 | 101.97 | 90.46 | 20.37 |
| 16 | - | 62.91 | 41.60 | 52.26 | 15.07 |
| 17 | 52.73 | 82.45 | 53.82 | 63.00 | 16.85 |
| 18 | - | 75.83 | - | 75.83 | N/A |

*Table 4: Distance from Drone to Sheep at Alert*

| Run No. | Repetition 1 (m) | Repetition 2 (m) | Repetition 3 (m) | Average (m) | Standard Deviation (m) |
| --- | --- | --- | --- | --- | --- |
| 1 | 28.44 | 74.58 | 12.35 | 38.46 | 32.31 |
| 2 | 28.80 | 32.01 | 27.64 | 29.48 | 2.27 |
| 3 | 38.35 | 33.19 | 115.39 | 62.31 | 46.04 |
| 4 | 24.57 | 55.92 | 42.97 | 41.16 | 15.75 |
| 5 | 46.57 | 30.91 | 31.43 | 36.30 | 8.89 |
| 6 | 35.85 | 39.04 | 33.40 | 36.10 | 2.83 |
| 7 | 11.32 | 32.21 | 73.70 | 39.08 | 31.75 |
| 8 | 44.72 | 24.41 | 32.59 | 33.91 | 10.22 |
| 9 | 42.16 | 107.09 | 76.40 | 75.22 | 32.48 |
| 10 | 13.36 | 46.75 | 80.52 | 46.88 | 33.58 |
| 11 | 37.34 | 79.84 | 134.66 | 83.95 | 48.79 |
| 12 | 30.77 | 61.51 | 61.46 | 51.24 | 17.73 |
| 13 | 16.38 | 32.87 | 90.77 | 46.67 | 39.07 |
| 14 | 33.99 | 83.95 | 44.70 | 54.21 | 26.30 |
| 15 | 30.36 | 54.37 | 73.02 | 52.58 | 21.39 |
| 16 | 57.55 | 33.02 | 27.94 | 39.50 | 15.84 |
| 17 | 40.82 | 57.00 | 24.98 | 40.94 | 16.01 |
| 18 | 88.70 | 40.40 | 95.92 | 75.01 | 30.18 |

*Table 5: Distance from Drone to Sheep at Initiating Drive*

| Statistics | Repetition 1 | Repetition 2 | Repetition 3 |
| --- | --- | --- | --- |
| Count | 18 | 18 | 18 |
| Sum | 2,622.0 | 2,494.0 | 2,490.0 |
| Mean | 145.667 | 138.556 | 138.333 |
| Median | 143.0 | 140.0 | 141.0 |
| Mode | 143.0 | 145.0 | 145.0 |
| Min | 122.0 | 113.0 | 91.0 |
| Max | 205.0 | 161.0 | 157.0 |
| Skewness | 1.8493 | -0.23753 | -1.9278 |
| Kurtosis | 4.7052 | -0.36023 | 5.5357 |
| Standard Deviation (Population) | 18.821 | 13.238 | 14.468 |
| Standard Deviation (Sample) | 19.366 | 13.622 | 14.888 |
| Variance (Population) | 354.222 | 175.247 | 209.333 |
| Variance (Sample) | 375.059 | 185.556 | 221.647 |
| Anderson Darling test for normality | | | |
| Value | 0.82592 | 0.96774 | 0.83702 |
| p-Value | **0.0036** | 0.7544 | **0.0053** |

*Table 6: Statistics and Normality Test for Heart Rate (beats/min) at Alert Distance*

| H_0_ (NULL) | | 'Dataset(k)' Median = 'Dataset(k+1)' Median | | | | | | | | |  |  |  |
| --- | --- | --- | --- | --- | --- | --- | --- | --- | --- | --- | --- | --- | --- |
| H_1_ (ALT) | | 'Dataset(k)' Median not equal to 'Dataset(k+1)' Median | | | | | | | | |  |  |  |
|  | | | Repetition 1 | | | Repetition 2 | | | Repetition 3 | | |  | |
| Repetition 1 | | |  | | |  | | |  | | |  | |
| Repetition 2 | | | Adj. p Value 0.3668  Test Stat = 362.0 | | |  | | |  | | |  | |
| Repetition 3 | | | Adj. p Value 0.476  Test Stat = 356.0 | | | Adj. p Value 0.8991  Test Stat = 328.5 | | |  | | |  | |
|  | |  | | |  | |  |  | | |  |  |  |
| H_0_ (NULL) | Variances are equal | | | | | | | | |  | | |  |
| H_1_ (ALT) | Variances are not equal | | | | | | | | |  |  |  |  |
| Sufficient evidence does not exist to conclude the Variances are different at alpha = 0.05 level. | | | | | | | | | | | | |  |
|  |  |  |  |  |  |  |  |  |  |  |  |  |  |
| p Value 0.8287 | | | | Test Statistic (L): 0.18861 | | | | | | | | |  |
|  |  |  |  | Statistics calculated using the Median. | | | | | |  |  |  |  |

*Table 7: Mann Whitney and Levene’s Tests of Heart Rate (beats/min) at Alert Distance between repetitions*

| Statistics | Repetition 1 | Repetition 2 | Repetition 3 |
| --- | --- | --- | --- |
| Count | 18 | 18 | 18 |
| Sum | 2,924.0 | 2,596.0 | 2,550.0 |
| Mean | 162.444 | 144.222 | 141.667 |
| Median | 157.0 | 145.0 | 143.5 |
| Mode | 143,149,189 | 145.0 | 149.0 |
| Min | 131.0 | 113.0 | 101.0 |
| Max | 211.0 | 169.0 | 167.0 |
| Skewness | 0.64667 | -0.14427 | -0.92365 |
| Kurtosis | -0.65165 | -0.22815 | 1.6469 |
| Standard Deviation (Population) | 23.655 | 14.695 | 14.955 |
| Standard Deviation (Sample) | 24.341 | 15.121 | 15.389 |
| Variance (Population) | 559.58 | 215.951 | 223.667 |
| Variance (Sample) | 592.497 | 228.654 | 236.824 |
| Anderson Darling test for normality | | | |
| Value | 0.92491 | 0.97514 | 0.95199 |
| p-Value | 0.1579 | 0.8867 | 0.4571 |

*Table 8: Statistics and Normality Test for Heart Rate (beats/min) at Distance Initiating Drive*

| H_0_ (NULL) | 'Dataset(k)' Median = 'Dataset(k+1)' Median | | |  |
| --- | --- | --- | --- | --- |
| H_1_ (ALT) | 'Dataset(k)' Median not equal to 'Dataset(k+1)' Median | | |  |
|  | Repetition 1 | Repetition 2 | Repetition 3 | |
| Repetition 1 |  |  |  | |
| Repetition 2 | p Value **0.0108**  Test Stat = 2.6979  CI = (4.496, 31.948) |  |  | |
| Repetition 3 | p Value **0.0043**  Test Stat = 3.0611  CI = (6.9834, 34.572) | p Value 0.6185  Test Stat = 0.50254  CI = (-7.7789, 12.89 |  | |

| H_0_ (NULL) | Dataset(k)' Variance = 'Dataset(k+1)' Variance | | |  |
| --- | --- | --- | --- | --- |
| H_1_ (ALT) | Dataset(k)' Variance not equal to 'Dataset(k+1)' Variance | | |  |
|  | Repetition 1 | Repetition 2 | Repetition 3 | |
| Repetition 1 |  |  |  | |
| Repetition 2 | p Value **0.0574**  Test Stat = 2.5912 |  |  | |
| Repetition 3 | p Value **0.0669**  Test Stat = 2.5018 | p Value 0.9431  Test Stat = 0.9655 |  | |

*Table 9: Two Sample t-Test and F-Test of Heart Rate (beats/min) at Distance Initiating Drive between repetitions*

| Statistics | Repetition 1 | Repetition 2 | Repetition 3 |
| --- | --- | --- | --- |
| Count | 15 | 11 | 12 |
| Sum | 984.385 | 845.311 | 936.631 |
| Mean | 65.626 | 76.846 | 78.053 |
| Median | 66.941 | 81.615 | 85.201 |
| Mode | NA | NA | NA |
| Min | 21.514 | 37.725 | 31.814 |
| Max | 107.57 | 102.452 | 135.824 |
| Skewness | 0.2068 | -0.87285 | 0.01146 |
| Kurtosis | -0.5664 | -0.013089 | -1.4849 |
| Standard Deviation (Population) | 25.513 | 19.733 | 33.936 |
| Standard Deviation (Sample) | 26.408 | 20.696 | 35.445 |
| Variance (Population) | 650.912 | 389.385 | 1,151.63 |
| Variance (Sample) | 697.405 | 428.324 | 1,256.32 |
| Anderson Darling test for normality | | | |
| Value | 0.32826 | 0.44494 | 0.47903 |
| p-Value | 0.4779 | 0.229 | 0.1901 |

*Table 10: Statistics and Normality Test for Alert Distance*

| H_0_ (NULL) | 'Dataset(k)' Median = 'Dataset(k+1)' Median | | |  |
| --- | --- | --- | --- | --- |
| H_1_ (ALT) | 'Dataset(k)' Median not equal to 'Dataset(k+1)' Median | | |  |
|  | Repetition 1 | Repetition 2 | Repetition 3 | |
| Repetition 1 |  |  |  | |
| Repetition 2 | p Value 0.2541  Test Stat = -1.1684  CI = (-31.041, 8.5999) |  |  | |
| Repetition 3 | p Value 0.3062  Test Stat = -1.0447  CI = (-36.926, 12.072) | p Value 0.9225  Test Stat = -0.0984  CI = (-26.693, 24.281) |  | |

| H_0_ (NULL) | Dataset(k)' Variance = 'Dataset(k+1)' Variance | | |  |
| --- | --- | --- | --- | --- |
| H_1_ (ALT) | Dataset(k)' Variance not equal to 'Dataset(k+1)' Variance | | |  |
|  | Repetition 1 | Repetition 2 | Repetition 3 | |
| Repetition 1 |  |  |  | |
| Repetition 2 | p Value 0.4425  Test Stat = 1.6282 |  |  | |
| Repetition 3 | p Value 0.2983  Test Stat = 0.55512 | p Value 0.101  Test Stat = 0.34093 |  | |

*Table 11: t-test and F-test of Alert Distance between repetitions*

| Statistics | Repetition 1 | Repetition 2 | Repetition 3 |
| --- | --- | --- | --- |
| Count | 18 | 18 | 18 |
| Sum | 650.034 | 919.09 | 1,079.84 |
| Mean | 36.113 | 51.061 | 59.991 |
| Median | 34.92 | 43.575 | 53.077 |
| Mode | NA | NA | NA |
| Min | 11.316 | 24.414 | 12.349 |
| Max | 88.699 | 107.09 | 134.662 |
| Skewness | 1.4387 | 1.0469 | 0.62668 |
| Kurtosis | 3.8698 | 0.48719 | -0.43454 |
| Standard Deviation (Population) | 17.172 | 22.197 | 33.582 |
| Standard Deviation (Sample) | 17.67 | 22.84 | 34.556 |
| Variance (Population) | 294.876 | 492.699 | 1,127.76 |
| Variance (Sample) | 312.221 | 521.681 | 1,194.09 |
| Anderson Darling test for normality | | | |
| Value | 0.61351 | 0.78236 | 0.50346 |
| p-Value | **0.0934** | **0.0341** | 0.178 |

*Table 12: Statistics and Normality Test for Distance of Initiating Drive*

| H_0_ (NULL) | | 'Dataset(k)' Median = 'Dataset(k+1)' Median | | | | | | | | |  |  |  |
| --- | --- | --- | --- | --- | --- | --- | --- | --- | --- | --- | --- | --- | --- |
| H_1_ (ALT) | | 'Dataset(k)' Median not equal to 'Dataset(k+1)' Median | | | | | | | | |  |  |  |
|  | | | Repetition 1 | | | Repetition 2 | | | Repetition 3 | | |  | |
| Repetition 1 | | |  | | |  | | |  | | |  | |
| Repetition 2 | | | p Value **0.048**  Test Stat = 270.0 | | |  | | |  | | |  | |
| Repetition 3 | | | p Value **0.0598**  Test Stat = 273.0 | | | p Value 0.6693  Test Stat = 319.0 | | |  | | |  | |
|  | |  | | |  | |  |  | | |  |  |  |
| H_0_ (NULL) | Variances are equal | | | | | | | | |  | | |  |
| H_1_ (ALT) | Variances are not equal | | | | | | | | |  |  |  |  |
| Based on this sample you can be 99.3% confident that Variances are different. | | | | | | | | | | | | |  |
|  |  |  |  |  |  |  |  |  |  |  |  |  |  |
| p Value 0.007 | | | | Test Statistic (L): 5.4716 | | | | | | | | |  |
|  |  |  |  | Statistics calculated using the Median. | | | | | |  |  |  |  |

*Table 13: Mann Whitney and Levene’s Tests of Distance Initiating Drive between repetitions*
